# Measuring particle charges in high electric fields of gas insulation systems using tracking velocimetry

**DOI:** 10.1038/s41598-025-18887-x

**Published:** 2025-10-07

**Authors:** Hans-Christoph Töpper, Christian M. Franck

**Affiliations:** Institute for Power Systems and High Voltage Technology, ETH Zurich, Zurich, Switzerland

**Keywords:** Gas insulation system, Electric charge, Particle tracking velocimetry, Electrical and electronic engineering, Energy infrastructure

## Abstract

The electric charges of particles are a decisive factor influencing their behavior in electric fields, particularly in high-voltage gas insulation systems. The performance of the latter can be significantly undermined by charged particles, which potentially cause equipment failure in the energy transmission system. This study presents a novel in-situ charge measurement approach using particle tracking velocimetry based on high-speed imaging. For the first time, charge polarities, magnitudes, and distributions are quantified in high electric fields of gas insulations. Characterizing metallic and dielectric particles covering a broad property spectrum allows for identifying decisive charge accumulation parameters. The results show that some particle materials exhibit no detectable charge, while others dynamically acquire broad charge distributions. Among the most influential parameters are the particle material density, the adhesive force between the particles and electrodes, and the applied electric field strength. Particle properties such as the electrical conductivity and relative permittivity appear negligible indicating the surface conductivity to be decisive. The observed minimum charge magnitudes align well with theoretical force-based expectations. Maximum charges, however, are not predictable using such approaches resulting in significant underestimations. Additionally, it is shown that the charge depends on the direction of the particle motion in the electric field. These findings validate the suitability and necessity of the developed measurement approach, highlight statistical charge variability, and inform our understanding of how metallic and dielectric particle dynamics influence the performance of gas insulation systems.

## Introduction

### Particles in gas-insulated switchgear

Gas-insulated substations are built with compact encapsulated switchgear consisting of high-voltage components such as circuit breakers, disconnectors, instrument transformers, or busbars. These systems ensure efficient and reliable operation of the electric power grid and are required for its further expansion^1,2^, given the currently advancing electrification^3^. The key performance measure of such insulation systems is the electrical withstand strength, characterizing the electric field magnitude at which the insulation gas transitions to a conducting state. Stressing systems past the withstand strength, causing an insulation breakdown, must be strictly avoided, as the equipment can be rendered inoperable due to internal arcs, which cause extremely high temperatures and rapidly increasing pressures.

Gas insulation systems have extensively been studied to achieve high withstand strengths and compact sizes. These studies quantified the influence of electrodes^4,5^, electrical stresses^6,7^, and a broad spectrum of insulation gas properties^8–10^. In addition, the presence of particles has been described as critically influencing insulation performance^11^. If they are not removed from the equipment before operation, they could even cause a breakdown of the insulation gas. Great care is taken to avoid their presence inside the equipment along all stages from manufacturing and assembly to maintenance in the field. However, due to their highly pervasive nature and small geometrical dimensions, such contamination cannot always be avoided and is challenging to detect and remove. This requires incorporating a sufficient safety margin in the equipment design limiting a further size reduction and efficiency increase of any gas insulation system.

The scientific literature quantifies the performance-diminishing effect of particles across various conditions. Spherical iron particles reduced the N$_2$ breakdown strength under AC stress by up to 25 %^12^. The breakdown strength of SF$_6$ was lowered by 30 % through spherical and wiry copper and aluminum particles under AC and DC stress^13^. An even higher performance-diminishing effect of approximately 60 % for SF$_6$ under AC stress was observed for iron particles^11^. The same decrease in the SF$_6$ performance has also been reported for brass particles under DC conditions ^13^. A similarly pronounced impact on gas insulation performances was observed for dielectric particles, though this remains rarely studied. Sand particles reduced the breakdown strength of air under AC stress by 12 %^13^ to up to 40 %^11^. Next to the particle-induced breakdowns, further particle-related phenomena potentially diminishing the insulation strength were observed. These include micro discharges^14^, ionic winds^15^, and the so-called firefly flight^14,16^. Additionally, extensive particle motions were observed^13,16–20^. The latter phenomena were reported to be influenced by parameters such as the pressure of the gas insulation ^21^, potentially used dielectric electrode coatings^18^, particle material type^22^, particle size^16^, and particle position in the system^17,20,23^ collectively highlighting complex dependencies.

Despite the diversity of the phenomena, they are all caused by one underlying commonality which is the electric charge of the particles obtained through contact with the electrodes in the high electric field. It is decisive to the gas insulation performance as it potentially causes localized field distortions, partial discharges, or initiates breakdowns. Understanding the charge dynamics and quantifying how the polarity, magnitude, and distribution of the charge are influenced by particle properties and electric field characteristics are therefore essential for formulating qualitative and quantitative physical charge models and assessing their impact on electric energy transmission systems.

### Characterization of electric particle charges in gas insulation systems

The electric charges of particles in electric fields of gas insulation systems remain scarcely studied as only the average charge magnitudes of a few metallic materials possessing diameters of a few millimeters were investigated. Experimentally, these charges have been determined by one of two techniques. An average charge was calculated by measuring the electric current between two electrodes due to charges transported by multiple particles. For aluminum wires, this resulted in approximately 300 pC to 3,000 pC per up and down cycle, depending on the electric field strength^13^. Alternatively, large particle quantities were collected in a Faraday cup connected to a coulombmeter. By this, the average aluminum particle charge was reported to be 50 pC to 150 pC^15^ and – 200 pC to 300 pC depending on the field strength^24^. For stainless-steel particles, – 235 pC to 250 pC was measured^17^.

Theoretical analyses of similarly sized metallic particles moving in electric fields of gas insulations assumed constant charges obtained through physical contact with the electrodes such as 140 pC and 240 pC for steel ^12^ or calculated them to be approximately 180 pC^24^ or 1,200 pC^25^ in the case of aluminum. The formulas reveal a notable diversity in the underlying assumptions, decisive particle characteristics, and proportionalities between particle charge and electric field strength. For instance, while several studies^17,19,26^ focus on spherical particles and took the particle radius as the only particle descriptor and applied empirical fitting presuming a linear proportionality between the particle charge and electric field strength, other approaches diverge significantly. Next to only accounting for the radius, an approach was described taking the weight as the defining particle property^27^. This was based on the assumption that the electric force resulting from the product of the charge and field strength compensates the gravitational force to cause particle motion, and it proposes an inverse proportionality between charge and electric field strength. In contrast, the particle surface area has also been used as a charge-defining property dismissing all other material characteristics^18^. Here, the electric field strength was integrated across the particle surface based on empirical fitting resulting in a linear proportionality between charge and field strength. Additionally, elongated particles were also described by incorporating the lengths of the two main axes^20,28,29^. This approach employed an empirical fitting method possessing a linear proportionality between the charge and field strength. While these studies possess some commonalities and enabled characterizations of metallic particles, there is no unified theoretical approach due to the considerable variability in how the charges have been calculated and most importantly what proportionality describes the dependency of the charge on the field strength.

Beyond gas insulation systems but within the field of electric energy transmission equipment, electric particle charges have been investigated in transformer oil^30^. Here, the charge of single stainless steel particles with diameters between 1 mm and 3.2 mm were determined based on their levitation, transferred charge between two electrodes, and by manually evaluating trajectories captured by high-speed imaging. Although this provides otherwise inaccessible quantitative charge data, the results cannot be transferred to particles in gas insulation systems as the dominant forces, particle diameters, and materials differ significantly. Additionally, charge statistics were not captured. Mostly, however, particle charges were determined in other contexts. Particularly, charges obtained through triboelectric effects, have been characterized across various dielectric materials. In bulk analyses accounting for statistical effects, powders were charged through physical contact with each other and then driven through uniform electric fields by gravity or forced airflow. For potato starch particles, this showed electrical charges in the range of – 200 fC to 200 fC^31^, – 34 nC to 85 nC for aerosols^32^, and charge-to-mass ratios of approximately 1 mC/kg to 10 mC/kg for minerals such as CaCO$_3$ ^33^ and MgCO$_3$ ^34^. The technique behind these characterizations was particle tracking velocimetry (PTV) which has been proven to be a versatile tool across multiple disciplines by enabling spatially resolved multi-dimensional tracking of objects in motion. Exemplary chosen analyses show that it supported the understanding and quantification of phenomena such as the motion of transformer oil^35,36^, metal spatter in powder bed fusion^37^, turbulent gas flows^38^, the motion of aerosolized particles^39^, bubbles^40^, and plasma actuator phenomena^41^. Across these phenomena, PTV proved suitable for tracking particles possessing diameters between the sub-micrometer scales^42^ and approximately a few hundred micrometers^37,39,43^. Practically, tracking is done by capturing the particle positions over time using one^40,42,44,45^ or multiple synchronized high-speed cameras^37,46^, including configurations with view splitters^47^. Illuminated by high-intensity light sources such as lasers^40,42,44,45,48^ and powerful LEDs^49^, the particle positions were subsequently evaluated using automated extraction algorithms.

### Novel charge measurement approach

While previous studies have demonstrated the significance of particles to gas-insulated switchgear and that metallic materials can acquire electric charges across a broad spectrum, the experimental techniques used for these characterizations have notable limitations. The methods do not measure the charge while the particles are present in the electric field of gas-insulated switchgear, simultaneously capture charges of multiple particles, or differentiate between motion directionalities. Additionally, the theoretical approaches relying on empirical fitting, mostly accounting for geometrical particle properties only, and showing different proportionalities between key characteristics were applied to a few metallic particles only. This offers the opportunity to develop and apply a novel measurement approach to compensate for these drawbacks. As the benefits provided by PTV in other fields are promising for understanding dynamic particle charge processes in gas insulation systems, it is chosen as the key method in this contribution.

For the first time in the context of gas insulation systems, individual particle charges can be quantified in-situ and non-intrusively by using high-speed imaging-based PTV and force-based motion calculations. Thereby, the charge polarity and magnitude of individual particles are quantified and charge distributions obtained across various particle materials and electric field strengths. The data is intended to support deriving physical models predicting particle charges based on their properties and the field strength, calculating field distortions due to the charge, assessing their criticality regarding the insulation performance, or evaluating if mitigation strategies are required in production. Due to the in-situ nature of the approach not interfering with the gas insulation system, unaltered particle charge data is obtained. These benefits are applicable across all electrode types, electrical stresses, and gas insulation materials as the approach applies to conventional high-voltage gas insulation test setups.

**Fig. 1:**
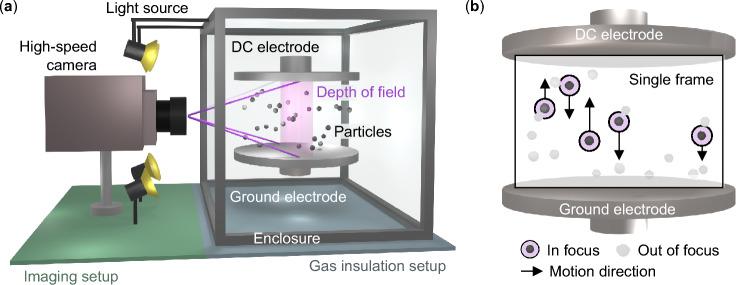
The schematic setup overview of the developed measurement approach is shown. **a** Inside a protective enclosure, two electrodes create a uniform DC field in which the particles are placed. Focused light sources provide sufficient illumination for the high-speed camera to capture the motion in the middle of the electric field. **b** Only the particles within the depth of field are visible. The individual high-speed camera frames show the positions of multiple particles simultaneously over time. These positions are used for the charge quantification.

The experimental setup of the developed approach integrating a high-speed imaging system with a laboratory-scale gas insulation experiment is schematically shown in Fig. 1. The camera captures the motion of the particles illuminated by focused light sources. Thereby, only the ones moving in a narrow depth of field in the middle of the electric field are visible. The latter is created between two plane electrodes of which the lower one is grounded and the upper one is connected to a high DC voltage.

The uniform DC field was chosen in this analysis as it eliminates frequency-dependent and dielectrophoretic effects potentially affecting the charge measurement. In this electric field, the particles are observed to move repeatedly up and down along the vertical field lines with resting times at the electrodes. The forces driving this motion are primarily the electric and gravitational forces. Thereby, the particles acquire a negative charge at the lower electrode, which is reversed upon contact with the upper electrode. By automatically quantifying the trajectories during the gap crossing, the acceleration of each particle can be determined. Therefore, multiple image processing techniques such as Gaussian smoothing and sub-pixel particle center detection are used. As the particle material density and diameter are known in addition to the electric field strength, the electric charge can be calculated based on the motion equation. This allows quantifying the influence of particle material characteristics, such as density, conductivity, and permittivity, on their charge accumulation potential. The developed approach is applied to fifteen different particle materials, both dielectric and metallic, across five electric field strengths.

## Results

### Statistics of electric particle charges

**Fig. 2:**
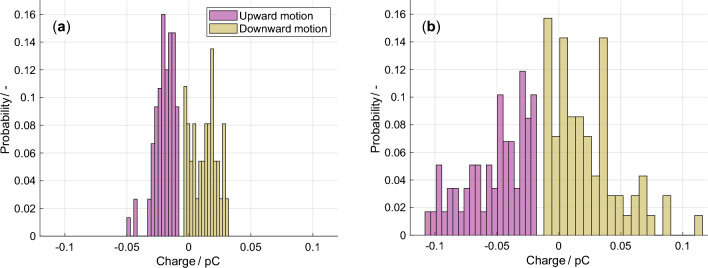
The particle charge distributions at 4 kV/cm in a uniform DC field are shown for two materials. **a** For vanadium particles, the charges of the particles moving upward and downward are shown. **b** Similarly, the charge distributions of zinc oxide particles are shown.

The measurement results show that the electric particle charges in a constant DC field possess a dynamic and statistical character. It is observed that particles can possess different charge polarities and a range of charge magnitudes necessitating a statistical approach to capture the underlying variability. Approximating the charge for a specific particle material and field strength by a single scalar value such as the mean or median value would underestimate occurring magnitudes by at least a factor of two since the distributions are broad and asymmetric. Across all particle materials acquiring charges at the investigated field strengths, such distributions are observed. Figure 2 shows the obtained charge distributions for one metallic and one dielectric particle material. The metallic particle material exhibits a charge spread of approximately 0.1 pC, while the dielectric particles possess a distribution approximately twice as wide. Within these distributions, particles moving upward are consistently negatively charged, whereas those moving downward display both positive and negative polarities, with a predominance of positive values. Notably, the distributions of the upward and downward-moving particles do not overlap. This shows that differentiating between motion directions is vital to charge investigations. Although all distributions show a slight incline towards lower charge magnitudes, this effect becomes significant only when extreme values are included. Additionally, the asymmetry of the distributions where charges for upward and downward-moving particles are not mirrored at the zero-charge line suggests a dynamic and asymmetric process governing the charge phenomena in the electric field.

### Influence of the particle material

**Fig. 3:**
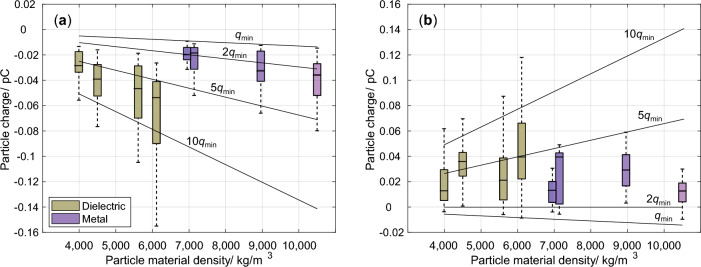
The measured and theoretically calculated charges are shown at an electric field strength of 4 kV/cm across all moving metallic and dielectric materials. **a** The upward-moving particles consistently possess negative charges. The theoretical charges range from an almost negligible ’overcharge’ to charges approximately ten times higher than the minimum charge compensating gravity. **b** Moving downward, the particles reverse their polarity except for a few minimum values. The charges fall in a similar range.

At a field strength of 4 kV/cm, some metallic and dielectric particles do not acquire sufficient charge for motion. These include calcium carbonate, lithium carbonate, silicone, zirconium silicate, iron, and titanium. Motion was observed for titanium dioxide but strong agglomeration occurred. In these cases covering both dielectric and metallic particles, the developed approach does not provide quantitative charge data. For the other materials investigated, however, motion is observed and electrical charges are measured. Figure 3 shows the obtained charges as a function of the particle material density for all moving dielectric and metallic materials. The median charge value is represented by the line within the boxes and their lower and upper ends mark the 1^st^ and 3^rd^ quartiles. The whiskers show the minimum and maximum values. In addition to the experimentally obtained values, theoretical particle charges are shown. As can be seen, their magnitudes range from below 10fC to approximately 150fC. The minimum and maximum absolute values for a given material type are separated by a factor of three to approximately a factor of ten. Regarding the ratio between the first and third charge quartiles, factors ranging between approximately two and three are observed. Regarding the magnitude of the absolute value and spread, the metallic particles mark the lower end, while the dielectric particles mark the upper.

Comparing the measurement results with calculated charges (cf. solid lines in Fig. 3), expressed as a multitude of the minimum charge $q_{\min }$, shows that they fall in a range of minimum charge multiples. $q_{\min }$ describes the minimum charge that is needed to force a particle motion against the gravitational force, cf. Eq. (11). Doubling this to $2q_{\min }$ relates to a charge that is twice as large as needed to overcome the gravitational force, $5q_{\min }$ five times higher, and so on. The reasons for this ’overcharging’ are elaborated further in the discussion section. This shows that the minimum absolute charge threshold for upward-moving particles is to overcome the gravitational force. For downward-moving particles, for which gravity acts in the direction of motion, the minimum requirement for a charge is very close to that for upward-moving particles, including the polarity. Interestingly, the theoretical minimum charge level of upward-moving particles is observed only for metallic particles, whereas the minimum charge for upward-moving dielectric particles is closer to $2q_{\min }$. The picture is different for downward-moving particles. For these, the minimum level is not as consistently observed for either material class, and values between $q_{\min }$ and 0pC are observed.

An influence of neither the relative permittivity for the dielectric particles varying by a factor of approximately fourteen nor the electrical conductivity covering more than 25 orders of magnitude across all materials, on the charge is observed (cf. Table 1). The density seems to be the most decisive intrinsic material parameter. Higher material densities are connected to higher charge magnitudes, whereby a distinction between the dielectric and metallic particles must be made. For dielectric particles, in general, higher charge amplitudes are observed.

### Influence of the electric field strength

**Fig. 4:**
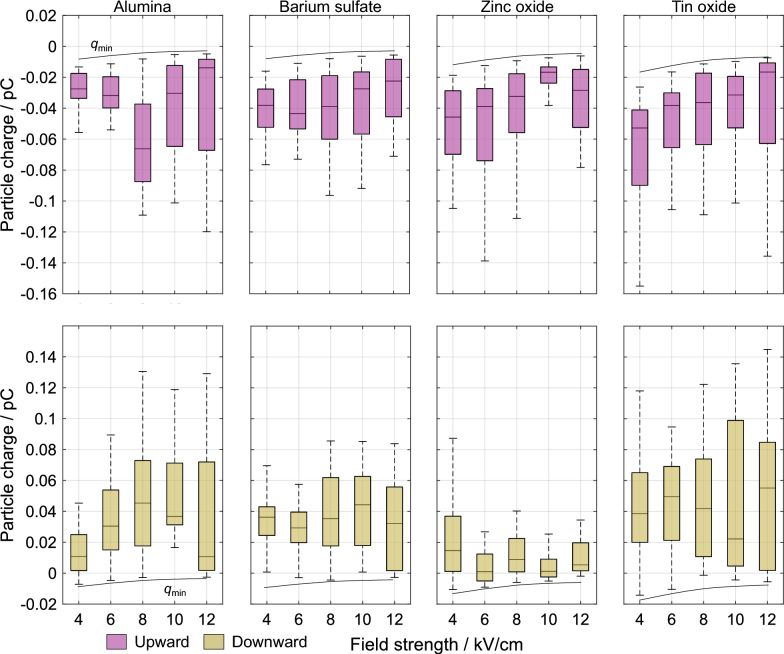
The electric charge is shown as a function of the electric field strength for dielectric particles. The material order from left to right is by an increasing material density. In the upper row, the charges of the upward-moving particles are shown while the lower row contains the charges of the downward-moving particles. The drawn lines represent the charge threshold based on a zero adhesive force.

Figure 4 shows the measured charges of four different dielectric materials for varying electric field strengths. Increasing the field strength from 4 kV/cm to 12 kV/cm for all materials exhibiting motion at the lower field shows that the magnitude, polarity, and distribution width of the charges remain largely unchanged. Among this, however, a pattern across all materials is observed. Increasing the field strength results in a decrease in the minimum charge magnitude of upward-moving particles. This occurs as only the particle properties and not the field strength variation determine the minimum electric force required for particle motion. At low adhesive forces relative to the gravitational force, an increase in the field strength requires less electric charge for an identical force. This effect becomes more pronounced at higher particle densities. Regarding the previously stated minimum charge magnitude dependent on the particle density, this shows that this threshold is extendable to various electric field strengths. This is also confirmed by by the minimum charge of downward-moving particles which possess adhesive forces close to zero, i.e. that particles moving downward do not possess charges lower than the minimum charge of the particle moving upward. Other parameters, such as relative permittivity, seem to have negligible influence.

**Fig. 5:**
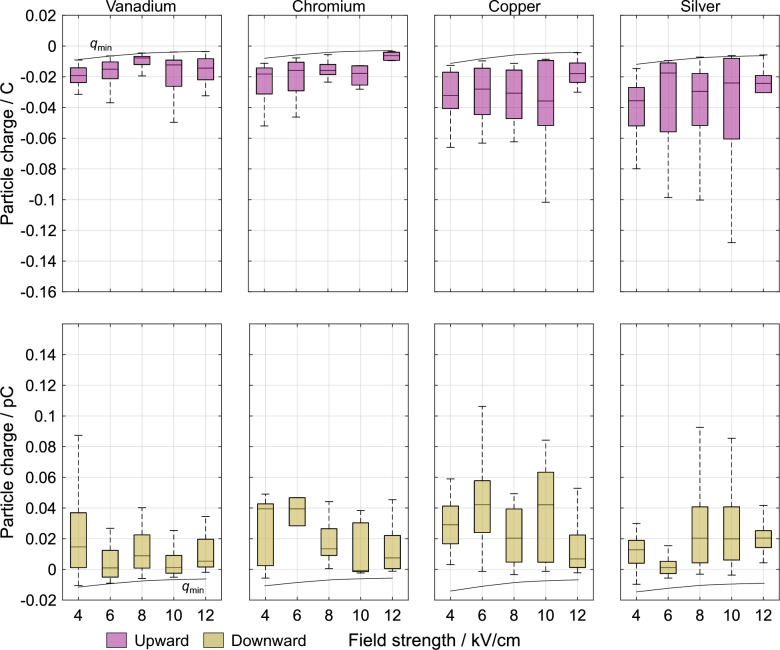
The measured electric charge is shown as a function of the electric field strength for metallic particles. The material order from left to right is by an increasing material density. The upper row contains the charge of the upward-moving particles and the lower row shows the charges of the downward-moving particles. The drawn lines for both motion directions show the charge threshold for an adhesive force of zero.

Figure 5 shows the obtained charge data of four different metallic materials for varying electric field strengths. Evaluating the charges of the metallic particles at the same field strengths shows identical observations. Hence, the force-based evaluation of the charge threshold seems independent of the particle material type. Two observable differences, however, are lower charge magnitudes and smaller charge spreads given for a specific material and field strength. This appears for the upward and downward motion. An influence of the electrical conductivities significantly higher than the ones of the dielectric materials is not identified.

It is interesting and important to note that the observed minimum charge shows the theoretically expected 1/*E* proportionality (cf. Eq. (14)), whereas the mean charge value does not.

## Discussion

The application of the developed measurement approach combining high-speed imaging, particle tracking velocimetry, and force-based charge calculations shows its capability to quantify individual particle charges in a gas insulation setup. Characterizing multiple particles of the same type at a given field strength simultaneously is necessary as statistical variability was observed across all investigated materials and field strengths. As the approach does not interfere with the particles and gas insulation system, the obtained charges can be considered unaltered by the measurement.

The algorithm applies to any particle motion captured in high-speed video recordings, provided the forces acting on the particles can be quantitatively described. This means that no changes would have to be made for the investigation of other uniform DC field configurations. However, non-uniform AC fields would require the time dependency of the electric field in addition to integrating dielectrophoretic forces. The effectiveness of the algorithm relies on the sub-pixel detection of the particle centers, which is essential given the typically limited resolution of high-speed cameras. While the current setup enables tracking of micrometer-sized particles, smaller particles or particles with less optical contrast may require adjustments. This could include aspects of the video processing, such as setting the initial brightness scaling and the parameters of the Gaussian smoothing. Additionally, the hardware, such as the illumination or focal length of the macro lens, might need to be adjusted. Since video processing parameters have to be chosen for each experimental setting, depending on the used hardware, a minimum detectable particle size is not strictly derivable.

Both dielectric and metallic particles possessing identical diameters, despite vastly different relative permittivity and electrical conductivities, acquire electric charges with mostly comparable magnitudes. The insensitivity of the charge amplitude with respect to the electrical conductivity suggests that the charging mechanism in electric fields of gas insulation systems is predominantly influenced by surface phenomena independent of bulk material properties. Adsorbed water, contaminants, or other environmental species often form a thin conductive layer allowing charge transfer at the surface independent of the electric conductivity of the particle^50^. The metallic materials frequently develop thin oxide or passivation layers possessing low conductivities consequently show a similar charge transfer behavior as they also possess such layers^51^.

As the set field strengths are not high enough to cause charge transfer due to field emission and the electrodes are at room temperature, ruling out phenomena such as thermionic effects, the charging seems to occur due to and only when there is physical contact between the particles and electrodes. This is supported by the observed constant accelerations of both upward-moving and downward-moving particles. Charge magnitude or polarity changes in the electric field during the gap crossing would be observable through changes in the acceleration. Driven by differences in work function, surface states, and local potential differences, the contact results in a gain or loss of electrons setting the particle charge polarity. This process, typically referred to as contact electrification, is inherently transient and localized. It is therefore not surprising that metallic particles, often assumed to dissipate charges rapidly, still exhibit significant charging under the experimental conditions. Information such as the time dependency of the charge transfer as a function of the material and field strength, however, is not obtainable by the approach.

The measured charges show that the forces acting on the particles offer explanations for the observed dynamics. For upward-moving particles, the electric force must overcome at least the gravitational force, but most likely also additional adhesive forces, which are known to be present in particular with small particles. This imposes a detachment threshold, ensuring only particles with sufficiently high charges move. Some particles may partially discharge without a polarity alteration and move downward under the influence of gravity. Others may remain attached to the upper electrode until they discharge and recharge with the opposite polarity. As soon as enough charge accumulates to overcome the adhesive forces, the downward motion occurs. This results in both charge polarities of downward-moving particles. Thus, the forces might collectively explain why upward-moving particles consistently possess the same charge polarity, whereas downward-moving particles show both polarities.

Since the adhesive forces often dominate the constant gravitational forces, variations in adhesion directly translate to a broad spectrum of required electrical forces for particle lift-off. The adhesive forces are possibly changing while the other forces remain constant for particles of similar size and density. This aligns with literature reports, strongly suggesting that the heterogeneity of adhesive interactions is a key driver of the broad charge distribution. Values for adhesive forces, typically reported for single particle sizes, can fluctuate by up to 200 % for rough and smooth surfaces^52^. This variation span can be even larger and cover two orders of magnitude^53^. Thereby, clear influential parameters are challenging to identify due to complex interactions^54^. Additionally, variations in the particle and electrode surface topographies can result in differences in the contact area. Aspects such as oxidation layers on the particle surfaces can locally influence the surface energy. Due to high particle velocities, local plastic deformation upon collision with the electrodes can occur and may influence the aforementioned aspects from one electrode gap crossing to another. Overall, these factors likely contribute to varying adhesive force values across otherwise similar particles.

Fast charging rates and lift-off delays caused by inertia might be another explanation for the observed ’overcharging’. However, as increasing particle material densities are not observed to cause higher charge values, the charging process seems to end once a particle detaches from the electrodes. This indicates that it is not overly charged due to the electron transfer happening much quicker compared to motion. Adhesion between the particle and electrode is thus assumed to be the dominating parameter to explain the broad charge spectrum observed under the conditions studied in this contribution.

A quantitative comparison of the results with experimental literature charge data is hardly possible. The transfer of charge measurements conducted in related components, such as high-voltage transformers filled with oil, is not possible due to significantly differing conditions, such as a higher viscosity of the medium surrounding the particles and potentially differing charging mechanisms. Mostly, however, much larger and only single metallic particles were studied^30^. Regarding gas insulations, however, the measured charges are approximately three orders of magnitude lower compared to published data^13,15,17,24^. This quantitative difference is expected as the particles investigated in the literature were approximately three orders of magnitude larger, and therefore heavier, requiring higher forces and thus electrical charges for their motion. Other influences such as adhesive forces potentially varying with the particle size, however, are not comparable. As the influence of parameters such as the electric field strength was not described, the obtained data offers new insights into the dependencies. The data also underlines the need to account for the statistics of the phenomenon. The latter was not provided by the described formulas resulting in scalar charges mostly dependent on particle geometries only^17–20,26,27,55^. Additionally, the direct proportionality between particle charge and electric field strength assumed by these formulas does not match the obtained results, as increasing the field strength by a factor of three does not result in a similar change in the mean, minimum, or maximum charge value. Not accurately capturing the latter might be critical to investigating the effect of the charge on the gas insulation performance. The highest charge amplitudes should be considered the most critical as they cause the highest field distortion and possibly release breakdown-initiating electrons into the field. Should breakdown investigations be conducted, those charges should be the focus.

The findings imply that metallic and dielectric particles are equally relevant to the performance of gaseous insulation systems if their ability to carry electrical charges is used as a measure of criticality. It is to be expected that the broad charge distributions obtained under defined laboratory conditions must also be expected under real-world conditions, and needs to be taken into account when estimating insulating performance. The electrically charged particles may lead to localized electric field enhancements, potentially causing breakdown-initiating discharges. These insights not only advance our understanding of particle charge dynamics but also hold practical implications for the design and maintenance of gas insulation systems. In particular, targeting a broader range of contaminants by tailoring surface coatings, and electrode materials, or incorporating additional particle filtering techniques during assembly and maintenance could enable a further size reduction and potentially overall reliability improvement of gas-insulated switchgear.

## Summary and conclusion

A novel in-situ charge measurement approach was developed, using particle tracking velocimetry based on high-speed imaging. For the first time, charge polarities, magnitudes, and distributions are quantified for various metallic and dielectric particles in high electric fields of gas insulation systems. The results show that some materials exhibit no detectable charge, while others dynamically acquire broad charge distributions. The particle material density, adhesive forces, and applied electric field strength are identified as the most influential parameters. A charged particle moves if the gravitational force and adhesive force are counteracted by the electric force which is set by the electric charge. Thereby, the minimum observed charge is proportional to the particle density and inversely proportional to the applied electric field strength. However, much higher charge magnitudes can occur too. Other particle properties such as the electrical conductivity and relative permittivity appear negligible. The particles can acquire charges that are up to a factor of 10 higher than the minimum required charges for particle motion. It is the latter particle charges that are most important when estimating the impact of the charged particles on the insulation’s withstand strength of gas-insulated systems, which will be the topic of a subsequent investigation.

## Methods

### Gas insulation setup and high-speed imaging

The uniform field-creating electrode configuration consists of two horizontally arranged polished steel Rogowski-type electrodes possessing a diameter of 80mm. The gap distance between them is set using a gauge block with a height of 10 ± 0,0005mm. The lower electrode is on ground potential and a positive DC voltage from 4 ± 0.05kV to 12 ± 0.05kV is applied to the upper electrode in 2 kV steps. The air between the electrodes is at ambient pressure, a temperature of $21 \pm 2\,^{\circ }\textrm{C}$, and a relative humidity of $55 \pm 3\,\%$ (H560 Dewpoint Pro, Dostmann). The electrode setup is enclosed in a plexiglass high-voltage safety box shielding it from potential disturbances such as air drafts. To ensure sufficient particle visibility, three focused halogen lights illuminate the electrode gap. The high-speed camera (Fastcam SA-Z High-Speed Camera, Photron) is equipped with a 100 mm macro lens (AT-X PRO D, 100 mm F2.8 Macro, Tokina) and observes the electrode gap at 20,000 frames per second. Both focus and aperture of the macro lens are set to create a narrow depth of field capturing only particles in the middle of the electric field. The resulting spatial pixel resolution is approximately 10 $\upmu$m by 10 $\upmu$m.

### Experimental procedure of particle positioning and field application

In the first step, and by using a grounded metal spatula, a few tens of particles of one material are manually placed and then evenly spaced on the lower grounded electrode in the section of the depth of field of the high-speed camera. Their large distance-to-diameter ratio minimizes mutual influences and avoids clustering once the field is applied. The initial charges potentially present are neutralized through contact with the spatula and grounded electrode. Measuring if triboelectric charging happened during the particle storage and handling, or if other mechanisms led to a net charge before the measurement, is therefore not necessary. Next to the positioning of the particles, closing the test circuit and initializing the high-speed camera add to the particles being in contact with the grounded electrode for multiple minutes. No voltage is applied during this step to avoid particle motion or charging. The positioning of individual particles in the middle of the electrode ensures that they are then only exposed to a uniform field.

Second, the positive DC voltage is applied to the upper electrode reaching its set values in a few milliseconds. The video recording begins based on a trigger approximately 100 milliseconds later to ensure steady-state particle motion observation. As multiple particles were placed evenly separated in the electric field, their motion occurred in parallel without interference. This allows for the extraction of statistical charge data from multiple independent particle trajectories.

Third, after capturing the individual trajectories for a set of particle material and field strength and disassembling the electrodes, the particles are removed and the electrode surfaces and the inside of the enclosure are cleaned using lint-free wipes (Science Precision Wipes, Kimtech) and a high-purity solvent (Acetone, Rotisolv, GC Ultra Grade, Carl Roth) to avoid cross-contamination. Since the particles stay intact during the experiment and do not leave residues on the polished electrode surfaces, this ensures identical conditions for all particles. After this step, the procedure begins again for the next particle material and electric field strength.

### Video processing and trajectory data extraction

**Fig. 6:**
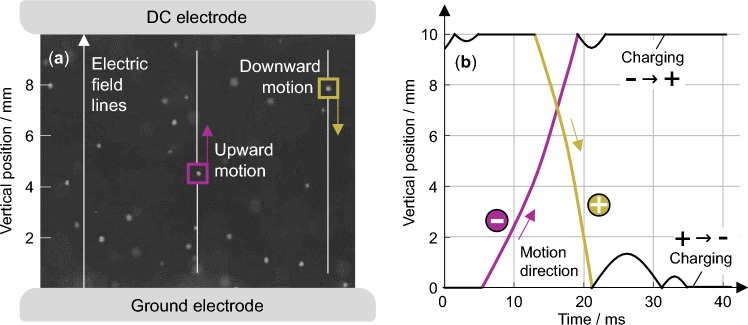
The motion of Al$_2$O$_3$ particles is shown at a field strength of 4 kV/cm. **a** Particles move independently in the electric field. Their size is slightly enlarged for enhanced visibility here. **b** The particle position is shown over time. A perpetual motion between the lower grounded and upper high-voltage electrode occurs. For the charge characterization, the acceleration during the gap crossing (colored) is automatically quantified. The observed bouncing and resting do not influence the charge quantification (black) and are not considered by the tracking algorithm. The charge polarity is indicated by a plus and a minus.

The high-speed camera images showing multiple particle positions simultaneously over time form the basis of the statistical particle charge characterization. The individual trajectorial data is automatically extracted for all particles simultaneously using particle tracking velocimetry implemented in MATLAB. Figure 6a shows an extract of an image from the high-speed camera video. The individual particles can be seen as white dots against the black background. This contrast results from the strong illumination of the halogen lights. They are observed to repeatedly move up and down along the vertical electric field lines. Figure 6b shows the particle position over time. After resting on the lower or upper electrode surface, where they acquire a negative or positive charge, they cross the gap. Only the motion during the gap crossing is used for the charge quantification. The resting on the electrode or bouncing upon collision is not considered.

First, each video frame is initially preprocessed to enhance particle visibility and isolate them from potential background noise. Therefore, a brightness scaling factor is applied to all pixels in the frame.

1 $$\begin{aligned} I_{\text {bright}}(x, y) = \min (I(x, y) \cdot f_{\text {brightness}}, 255) \end{aligned}$$

where $I(x, y)$ is the original pixel intensity at the position $(x, y)$ in the frame, $f_{\text {brightness}}$ is an adjustment factor of approximately 2 to 4, and $I_{\text {bright}}(x, y)$ is the intensity of the brightened pixel. **255** refers to the maximum pixel brightness which can be exceeded by applying the brightness factor to already bright areas of the frame. This is followed by two-dimensional Gaussian smoothing through convolution reducing the high-frequency noise in each frame.

2 $$\begin{aligned} I_{\text {smooth}}(x, y) = I_{\text {bright}}(x, y) * G_{\sigma }(x, y) \end{aligned}$$

where $G_{\sigma }(x, y)$ is a Gaussian kernel with the standard deviation $\sigma$ (set in the range between 1 and 1.5 and $I_{\text {smooth}}(x, y)$ the smoothed intensity of the pixels. A binary threshold technique based on morphological operations segments the particles from the background where the threshold value was chosen to both isolate particles and minimize noise. This threshold depends on how strongly the particle reflects the light and divides each image into two classes of which one contains the particles and the other noise that is to be removed.

3 $$\begin{aligned} \text {M}(I_{\text {smooth}}(x,y)) = {\left\{ \begin{array}{ll} 1, & \text {if } S(C) > T_{\text {threshold}} \text { for component } C, \\ 0, & \text {if } S(C) < T_{\text {threshold}} \text { for component } C \end{array}\right. } \end{aligned}$$

where $\text {M}(I_{\text {smooth}}(x,y))$ indicates if a pixel belongs to a particle or not as the morphological binarization output, *C* represents components in the image possibly showing a particle, *S*(*C*) is the number of pixels in the connected component, and $T_{\text {threshold}}$ the noise threshold (set in the range of 1–1.5). Only the pixels belonging to particles are further processed.

**Fig. 7:**
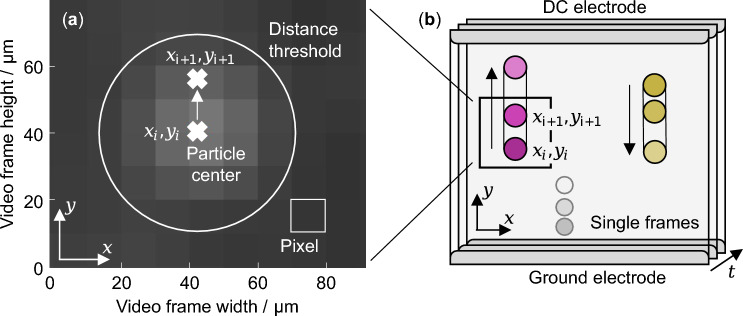
The algorithm functionality is shown based on Al$_2$O$_3$ particles. **a** A zoom-in of a high-speed camera frame is shown. The *x* and *y* positions of the particle centers at the time step *i* are automatically detected with sub-pixel accuracy. If the center does not move beyond the inter-frame distance threshold shown by the white circle in the next frame at the time step *i+1*, it is assigned to the same trajectory. **b** Connecting the particle positions across consecutive frames based on this detection method and the inter-frame particle distance threshold allows the reconstruction of the trajectories. This is shown schematically for one upward-moving and one downward-moving particle. A particle also present in the frames and detected with sub-pixel accuracy, but not assigned to these two trajectories, is shown in gray.

In the next step and for each set of pixels belonging to a particle, the particle centers are detected with sub-pixel accuracy. This is achieved by calculating the weighted pixel intensity centroid. This way, particle positions on the frames can accurately be determined despite covering only few pixels. This is key to the algorithm functionality, as high-speed cameras typically do not have high resolutions. The result of such a particle center detection is shown in Fig. 7a. The pixels illuminated beyond the actual particle diameter result from the Gaussian smoothing.

4 $$\begin{aligned} x_c = \frac{\sum x \cdot I_{\text {smooth}}(x,y)}{\sum I_{\text {smooth}}(x,y)} \end{aligned}$$

and

5 $$\begin{aligned} y_c = \frac{\sum y \cdot I_{\text {smooth}}(x,y)}{\sum I_{\text {smooth}}(x,y)} \end{aligned}$$

where *x* and *y* are the coordinates of each pixel, $I_{\text {smooth}}(x,y)$ is the intensity value of each pixel, and $x_c$ and $y_c$ are the particle centroids. Based on the first particle appearances, their trajectories are initiated.

6 $$\begin{aligned} T_i = C_i \end{aligned}$$

where $C_i$ is the centroid of the i-th particle and $T_i$ is the corresponding trajectory. Matching centroids into trajectories across consecutive frames is based on their Euclidean distance *d* across frames:

7 $$\begin{aligned} d = \sqrt{(x_{c,t} - x_{c,t-1})^2 + (y_{c,t} - y_{c,t-1})^2} \end{aligned}$$

where $x_{c,t}, y_{c,t}$ mark the position of a centroid at frame *t*, $x_{c,t-1}, y_{c,t-1}$ mark the position of the matching centroid at the previous frame $t-1$, and *d* is the distance between the centroids. If $d < d_{\text {threshold}}$, the position is added to the existing trajectory where $d_{\text {threshold}}$ (set in the range from 1 to 5 pixels) is the distance threshold below which centroids are considered a match. This results in the following expression and is schematically shown for three particle positions in Fig. 7b. The colored positions can be assigned to the trajectories while the positions shown in grey below to other particles.

8 $$\begin{aligned} T_j = T_j \cup \{ C_{\text {matched}} \} \end{aligned}$$

where $T_j$ marks the trajectory of the j-th particle and $C_\textrm{matched}$ the centroid that has been matched to the existing trajectory $T_j$. New trajectories are initiated for centroids that could not be matched to existing ones. To eliminate obtained trajectories not suited for further processing, only trajectories with a minimum number of points (set in the range from 50 to 100) providing sufficient data for accurate kinematic calculations are stored. The sub-pixel particle center detection and inter-frame distance threshold used for the trajectory reconstruction are shown in Fig. 7.

**Fig. 8:**
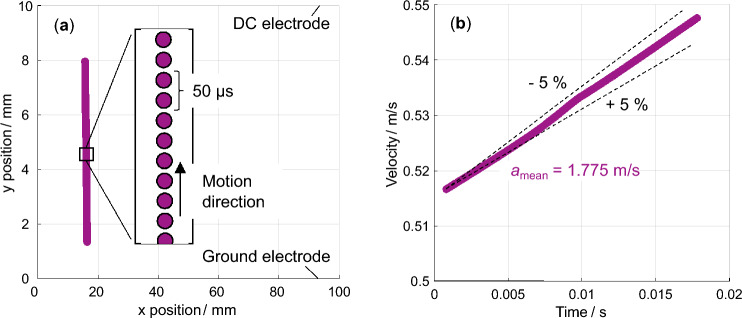
The trajectorial particle data provided by the tracking algorithm are shown. **a** The x and y positions of a single particle trajectory are shown during the gap crossing between the lower grounded and upper DC electrode. The zoom-in shows individual positions separated by the frame rate of the high-speed camera. **b** The particle velocity of the same trajectory shown as a function of time is linear. This shows an almost constant acceleration. The mean acceleration value along the path is significantly more stable compared to a $\pm 5\%$ corridor.

In the next step, the pixel coordinates of each trajectory are converted into meters based on the geometrical dimensions of the real-world section captured in the frame. This is required for the subsequent velocity and acceleration calculation in SI units. Based on the rate of change of position, the velocity is calculated, and based on the rate of velocity change, the acceleration is computed. The latter can be considered constant as an almost perfectly linear dependency of the particle velocity on the time is observed across all particle materials and field strengths independent of the motion direction. For a single particle, Fig. 8a shows the extracted positions during its upward motion along the vertical field lines from the lower to the upper electrode. On the time scale, the individual positions are separated by 50$\upmu$s due to the high-speed camera frame rate. This is shown by the zoom-in in which the motion direction is also indicated. Figure 8b shows the corresponding velocity over time calculated based on the time intervals between the individual positions. The corresponding acceleration can be considered constant.

The algorithm, particularly due to the sub-pixel particle center detection, has proven to work reliably and no further filtering of the obtained velocities and accelerations is needed. To differentiate between upward- and downward-moving particles, the trajectory sections are divided into two respective categories. A trajectory is classified as ‘upward’ if the particle consistently moves in the positive vertical direction whereas it is classified as ‘downward’ if the particle consistently moves in the negative vertical direction. As a result, quantified particle motion paths are obtained. Based on these, the particle charges are calculated using the forces acting on them during motion.

### Motion condition and calculation of electric charge

For particle motion to occur in a uniform electric DC field (cf. Fig. 6a), the net force determined by gravitational, adhesive, and electric force, needs to be positive and directed away from the electrode surface. Thereby, a distinction must be made between the lift-off from the lower electrode, where gravity must be compensated, and the upper electrode, where gravity assists detachment. As a result, the lift-off condition from the electrodes can generally be described as follows. The positive sign of the gravitational force describes lift-off from the lower electrode, the negative sign the lift-off from the upper electrode.

9 $$\begin{aligned} F_{\text {electric, lift-off}} = F_{\text {adhesion}} \pm F_{\text {gravitational}} \end{aligned}$$

$F_{\text {electric, lift-off}}$ is the electric force causing particle motion, $F_{\text {adhesion}}$ the adhesive force between particles and electrodes, and $F_{\text {gravitational}}$ the gravitational force of the particles. Expressing the lift-off electric force as the product of the electric field strength $E$ and particle lift-off charge $q_{\text {lift-off}}$ and the gravitational force as the product of the particle mass $m$ and gravitational constant $g$, and solving this for the lift-off charge results in the following expression. For the lower electrode, it shows that a particle charge equal to the sum of the adhesive and gravitational force divided by the electric field, it begins to move. For the upper electrode, the negative sign of the gravitational force applies.

10 $$\begin{aligned} q_{\text {lift-off}} = \frac{F_{\text {adhesion}} \pm m \cdot g}{E} \end{aligned}$$

As the particle charge is not observed to change after lift-off as it possesses a constant acceleration (cf. Fig. 8b) along the entire path between the electrodes, it is identical to the particle charge $q$ during motion (cf. Fig. 6b). The formula shows that adhesive forces are directly proportional to the charges required for motion. Consequently, particle charges higher than calculated based on the gravitational force can occur. If the adhesion between the particles and electrode is assumed to be zero, the following expression results for the absolute value of the minimum charge $q_{\text {min}}$ causing lift-off.

11 $$\begin{aligned} q_{\text {min}} = \left| \frac{\pm m \cdot g}{E} \right| = \frac{\rho \cdot \frac{4}{3} \pi r^3 \cdot g}{E} \end{aligned}$$

After lift-off, the forces acting on the moving particles can be described as follows. They are the basis for the charge characterization. The adhesion is not part of the equation as it only determines the lift-off threshold.

12 $$\begin{aligned} F_{\text {net}} = F_{\text {electric}} \pm F_{\text {gravitational}} + F_{\text {drag}} \end{aligned}$$

$F_{\text {net}}$ is the net force, $F_{\text {electric}}$ the driving electric force, and $F_{\text {gravitational}}$ adding to the net force during downward motion and reducing it during upward motion, and $F_{\text {drag}}$ is the drag force exerted by the gas acting. Accounting for the particle mass $m$, acceleration $a$, electric charge $q$, electric field strength $E$, gravitational constant $g$, dynamic viscosity of air $\eta$ of approximately $1.81 \cdot 10^{-5} \,\text {Pa} \cdot \text {s}$, particle radius $r$, and particle velocity $v$, it can be written:

13 $$\begin{aligned} m \cdot a = q \cdot E \pm m \cdot g - 6 \pi \eta r v \end{aligned}$$

Here, the drag force is approximated by Stokes’ law. This is common for particles, however, the influence of this term is negligible given the relatively low velocities. As shown, a decrease in the acceleration is not observed for the electrode gap crossing (cf. Fig. 8b). As this expression is universally applicable to particles in electric fields, it has already been applied in a variety of analyses^11,13,15^.

Solving this for the charge while distinguishing between the upward and downward motion, the following expression is obtained. This too has already been used in this form or slightly adapted in particle analyses in high-voltage equipment contexts^17,20,30^. The relative weight of each term, however, strongly depends on the specific circumstances, which limits the direct transfers between experiments. No unknowns remain in this equation. The particle density is given by the material type, the viscosity of air is known, the particle radius and electric field strength are set, and the trajectorial characteristics acceleration and velocity are provided by the tracking algorithm. Therefore, the particle charge can be calculated. Given the direction of the forces, however, the upward-moving and downward-moving particles must be handled separately.

14 $$\begin{aligned} q_{\text {up/down}} = \frac{\rho \cdot \frac{4}{3} \pi r^3 \cdot (a \pm g) + 6 \pi \eta r v}{E} \end{aligned}$$

$q_{\text {up}}$ is the charge of an upward-moving particle, $q_{\text {down}}$ is the charge of a downward-moving particle. The positive sign of the gravitational constant relates to the upward motion and the negative sign to the downward motion. Given a measured particle acceleration constant well below $\pm 5\%$ results in a charge uncertainty also well below $\pm 5\%$. Across all experimental parameters, the air viscosity is assumed to be constant and the drag force is observed to be significantly lower than the gravitational force. The drag force can thus also be neglected and Eq. (14) then shows the direct proportionality between the absolute electric charge and the material density of the particle. Additionally, it describes the inverse proportionality between the charge and electric field strength. As gravity must be compensated for upward motion, all accelerations result in the same charge polarity. For downward-moving particles, accelerations lower than the gravitational acceleration result in an opposite polarity charge compared to the particles possessing accelerations larger than the gravitational acceleration. The particle characteristics and electric field strength are given by the chosen experimental conditions. This leaves only the acceleration unknown which is quantified for the individual particles by the velocimetry algorithm.

### Metallic and dielectric particle materials

Fifteen metallic and dielectric particle materials possessing diameters of $(50 \pm 5) \, \upmu \text {m}$ and a maximum length-to-width aspect ratio of approximately 1.10 were investigated. They were chosen to represent materials potentially encountered in manufacturing and assembly of gas insulation systems, based on their prevalence in environmental dust, and to cover a wide range of relative permittivity, electrical conductivity, and mass density. Additionally, only particle types not harmful to human health were investigated, which is often not the case in these size ranges. Table 1 provides the particle material overview. They are all commercially available with the described particle diameter uncertainty specified by the manufacturers (Merck KGaA, Zest Dental Solutions, NanoChemazone, Stanford Advanced Materials). Their intrinsic material properties were taken from the materials databases PubChem^56^ and The Materials Project^57^. For the size range and shape, light microscopy showed that they possess shapes relatively spherical in all cases. As several factors such as surface effects, moisture adsorption, temperature fluctuations, or trapped charge effects influence even laboratory-grade materials, ranges are given for some materials. These ranges do not contribute to increased measurement uncertainties.

**Table 1:** Overview of the investigated metallic and dielectric particle materials. Their type, name, chemical formula, and physical properties relevant to their electric field interaction and electron mobility are listed. The * marks the particle materials for which no detectable charge was observed. In these cases, the particles did not depart from the lower electrode. This indicates an insufficient charge accumulation to overcome adhesion and gravity. As a result, the charge quantification through the motion-based algorithm was not possible. However, this does not mean that their charge was not influenced by the electric field. Materials for which an agglomeration of moving particles was observed are marked by **. In the latter case, a charge sufficient for motion is observed, however, the developed method only applies to individually moving particles.

Type	Name and chemical formula	Density in $\text {kg/m}^3$	Rel. permittivity	Conductivity in S/m
	Alumina $\text {Al}_2\text {O}_3$	3990	10	$10^{-18} - 10^{-16}$
	Barium sulfate $\text {BaSO}_4$	4500	11.4	$10^{-20} - 10^{-18}$
	Calcium carbonate* $\text {CaCO}_3$	2710	6.5	$10^{-18} - 10^{-16}$
	Lithium carbonate* $\text {Li}_2\text {CO}_3$	2110	6.5	$10^{-18} - 10^{-16}$
	Silicone* Si	2330	11.7	$10^{-5} - 10^{-4}$
	Tin oxide $\text {SnO}_2$	6950	23.4	$10^{-6} - 10^{-4}$
	Titanium dioxide** $\text {TiO}_2$	4230	78	$10^{-18} - 10^{-16}$
	Zinc oxide ZnO	5610	8.5	$10^{-10} - 10^{-6}$
Dielectric	Zirconium silicate* $\text {ZrSiO}_4$	4560	5.6	$10^{-18} - 10^{-16}$
	Silver Ag	10,500		$67 \times 10^6$
	Chromium Cr	7140		$7.9\times 10^6$
	Copper Cu	8960		$64 \times 10^6$
	Iron* Fe	7870		$11 \times 10^6$
	Titanium* Ti	4510		$2.3 \times 10^6$
Metallic	Vanadium V	6110	Not applicable	$5 \times 10^6$

## Data Availability

The code for the particle tracking velocimetry and charge calculation as well as the captured high-speed camera videos are available from the corresponding author on reasonable request.
